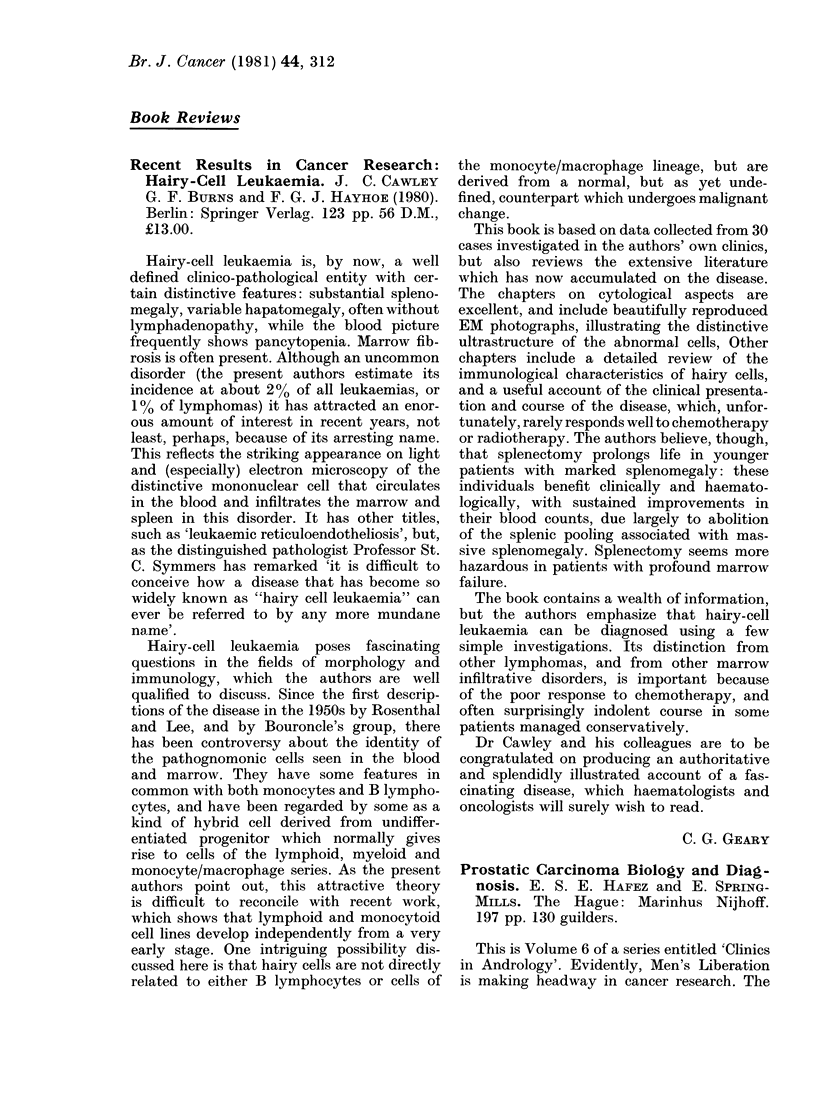# Recent Results in Cancer Research: Hairy-Cell Leukaemia

**Published:** 1981-08

**Authors:** C. G. Geary


### *Book Reviews*

**Recent Results in Cancer Research: Hairy-Cell Leukaemia.** J. C. Cawley G. F. Burns and F. G. J. Hayhoe (1980). Berlin: Springer Verlag. 123 pp. 56 D.M., £13.00.

Hairy-cell leukaemia is, by now, a well defined clinico-pathological entity with certain distinctive features: substantial splenomegaly, variable hapatomegaly, often without lymphadenopathy, while the blood picture frequently shows pancytopenia. Marrow fibrosis is often present. Although an uncommon disorder (the present authors estimate its incidence at about 2% of all leukaemias, or 1% of lymphomas) it has attracted an enorous amount of interest in recent years, not least, perhaps, because of its arresting name. This reflects the striking appearance on light and (especially) electron microscopy of the distinctive mononuclear cell that circulates in the blood and infiltrates the marrow and spleen in this disorder. It has other titles, such as 'leukaemic reticuloendotheliosis', but, as the distinguished pathologist Professor St. C. Symmers has remarked 'it is difficult to conceive how a disease that has become so widely known as "hairy cell leukaemia" can ever be referred to by any more mundane name'.

Hairy-cell leukaemia poses fascinating questions in the fields of morphology and immunology, which the authors are well qualified to discuss. Since the first descriptions of the disease in the 1950s by Rosenthal and Lee, and by Bouroncle's group, there has been controversy about the identity of the pathognomonic cells seen in the blood and marrow. They have some features in common with both monocytes and B lymphocytes, and have been regarded by some as a kind of hybrid cell derived from undifferentiated progenitor which normally gives rise to cells of the lymphoid, myeloid and monocyte/macrophage series. As the present authors point out, this attractive theory is difficult to reconcile with recent work, which shows that lymphoid and monocytoid cell lines develop independently from a very early stage. One intriguing possibility discussed here is that hairy cells are not directly related to either B lymphocytes or cells of the monocyte/macrophage lineage, but are derived from a normal, but as yet undefined, counterpart which undergoes malignant change.

This book is based on data collected from 30 cases investigated in the authors' own clinics, but also reviews the extensive literature which has now accumulated on the disease. The chapters on cytological aspects are excellent, and include beautifully reproduced EM photographs, illustrating the distinctive ultrastructure of the abnormal cells, Other chapters include a detailed review of the immunological characteristics of hairy cells, and a useful account of the clinical presentation and course of the disease, which, unfortunately, rarely responds well to chemotherapy or radiotherapy. The authors believe, though, that splenectomy prolongs life in younger patients with marked splenomegaly: these individuals benefit clinically and haematologically, with sustained improvements in their blood counts, due largely to abolition of the splenic pooling associated with massive splenomegaly. Splenectomy seems more hazardous in patients with profound marrow failure.

The book contains a wealth of information, but the authors emphasize that hairy-cell leukaemia can be diagnosed using a few simple investigations. Its distinction from other lymphomas, and from other marrow infiltrative disorders, is important because of the poor response to chemotherapy, and often surprisingly indolent course in some patients managed conservatively.

Dr Cawley and his colleagues are to be congratulated on producing an authoritative and splendidly illustrated account of a fascinating disease, which haematologists and oncologists will surely wish to read.

C. G. Geary